# Demonstration before the Georgia Surgeons’ Club

**Published:** 1913-12

**Authors:** W. F. Shallenberger, Montague L. Boyd


### DEMONSTRATION BEFORE THE GEORGIA SURGEONS' CLUB.

By Dr. W. F. Shallenberger and

Dr. Montague L. Boyd.

Dr. Shallenberger showed a woman who had had a chronic cystitis that had been cleared up under treatment. On this patient he demonstrated the method of female cystoscopy with the open or Kelly cystoscope. He showed all the details of the preparation of the patient, the cleaning up, the cocainization of the urethra, etc. Then the various steps in the cystoscopic examination were carried out and explained and the visiting men were given an opportunity of examining the bladder.

A ureteral catheter was prepared and the method of catheterizing the ureter, through the open cystoscope, was demonstrated. With the catheter in the left ureter the various uses of the ureteral catheter as a means for diagnosis and treatment were explained. It was also shown how the urine could be collected transvesically from the side that had not been catheterized thus making possible a differential functional test without the necessity of catheterizing both ureters.

Some X-ray plates showing collargol injections of the ureter and kidney pelvis were shown.

Dr. Boyd gave a cystoscopic demonstration on a patient with slight hypertrophy of the middle and lateral lobes of the prostate, with the method of the preparation of the patient for such an examination. The method of recording the findings was also shown.

Further, a demonstration was made of the phenolsulphonephthalein test for determining the functional capacity of the kidneys or kidney, with the method of reading the amount of the dye eliminated, and an explanation of the significance of a decrease output. In this connection a patient was shown who had had stones in both kidneys in whom there had been a very marked destruction of the kidney substance. The stones were removed and the patient was living a pretty normal life with only about one-fourth of the functioning part of the kidneys remaining.

---

### GEORGIA SURGEONS' CLUB.

OFFICERS.—E. C. Davis, M. D., Atlanta, Ga., President; Thos. J. McArthur, M. D., Cordele Ga., Vice-President; R. M. Harbin, M. D., Rome, Ga., Sec.-Treas.

EXECUTIVE COMMITTEE.—W. S. Goldsmith, M. D., Atlanta, Ga., Chairman; G. R. White, M. D., Savannah, Ga.; W. W. Battey, Jr., Augusta, Ga.

Foreword.—The purpose of the Georgia Surgeons' Club is to arrange in advance, through an Executive Committee, with the heads of the various clinics a program that will occupy the entire time allotted so that a short period of clinical study of surgery may be of value and furnish complete information as to transportation, hotels, etc., and the advantages thus offered will tempt surgeons whose limited time would otherwise forego clinical study hours.

It goes without saying that every surgical master would stand ready to extend courtesies to a body of earnest men if he has been duly apprised in a formal way.

### By-Laws Georgia Surgeons' Club.

#### ARTICLE I.

Names Georgia Surgeons' Club.

#### ARTICLE II.

Objects To raise the standard of surgery and hospitals in Georgia and furnish facilities for the study of clinical surgery

#### ARTICLE III.

Officerss Sec. 1. President, Vice-President, Secretary-Treasurer, and an Executive Committee of three, the officers being ex-officio members of this committee, all of them elected biennially except the Secretary-Treasurer, whose term of office shall be four years, and shall serve until their successors are elected.

Sec. 2. The executive powers of the Club shall be vested in the Executive Committee, subject to overrulement by a majority vote of the members at each regular meeting.

#### ARTICLE IV.

Members: Sec. 1. The qualifications for membership in the Medical Association of Georgia.

Sec. 2. Honorary members, without voting privileges, may be elected from other states and countries and from the Army, Navy and Marine Hospital Services by the Executive Committee.

#### ARTICLE V.

Meeting: Meetings of the Club shall be held in such cities as have suitable hospital facilities and at such time and place as may be determined by the Executive Committee, and any meeting after thirty days' notice by the Secretary and authorized by the Executive Committee shall constitute a regular meeting.

ARTICLE VI.

FEES At each meeting of the Club the membership in attendance only, shall be assessed such an amount as becomes necessary to meet current expenses.

